# Empirical Support for the Tenets of Sport Participation and Physical Activity-Based Models: A Scoping Review

**DOI:** 10.3389/fspor.2021.741495

**Published:** 2021-10-14

**Authors:** François Gallant, Mathieu Bélanger

**Affiliations:** ^1^Faculty of Medicine and Health Sciences, Université de Sherbrooke, Sherbrooke, QC, Canada; ^2^Centre de Formation Médicale du Nouveau-Brunswick, Moncton, NB, Canada; ^3^Vitalité Health Network, Moncton, NB, Canada

**Keywords:** athlete development models, scoping review, sport participation, tenet evaluation, sport participation and physical activity-based model

## Abstract

Notwithstanding their wide-spread use, it is unclear what level of empirical evidence exists to support sport participation and physical activity-based models. Sport participation and physical activity-based models characterize different stages of sport involvement based on sport activities (organized and unorganized) individuals take part in throughout their lifespan. The objectives of this scoping review was to explore the nature of empirical support for tenets of sport participation and physical activity-based models describing the evolution of an individuals' sport participation. Seventeen different sport participation models were identified through an iterative literature review, using a snowball search strategy and expert (*n* = 8) consultation. Of the identified models, three described the evolution of an individual's sport participation based on their participation in different activities at various stages of sport involvement and were retained for the review. A second literature review identified peer-reviewed publications supporting at least one tenet of these three models. Many tenets of retained models received some empirical support from some of the 38 publications identified, but some tenets were not tested. Most of the evidence supporting tenets originated from studies among elite-level athletes. Whereas some evidence exists to support current sport participation and physical activity models, more research is warranted, particularly among the general population of non-elite athletes, for the models to be used in full confidence to guide sport policies, programs, and practices.

## Introduction

Several authors have expressed that sport participation research is held back by insufficient details on the characterization of sport involvement of participants (Agans and Geldhof, 2012; Coalter, 2015; Evans et al., 2017; Robertson et al., 2019; Mosher et al., 2020). Generally speaking, studies often dichotomize sport involvement as participant/non-participant (Coalter, 2015; Robertson et al., 2019), which oversimplifies sport participation which could also account for type of sport, competitive level, length of involvement, role on the team, etc. (Robertson et al., 2019). Given the importance of sport participation for improved physical (Hebert et al., 2015, 2016) and mental health (Eime et al., 2013; Evans et al., 2017), there is a need to improve the characterization of sport involvement in the general population. This could increase understanding of the development of sport participation by providing insight into patterns associated with long-term involvement in sport and physical activity. Such improvements in understanding of sport participation development may be important for effective policy and planning around sport participation and delivery, especially when describing sport participation in a large number of individuals.

Multiple frameworks for understanding sport involvement have been suggested (e.g., sport participation models). Since national sport organizations across the globe are known to use sport participation models to guide their practices (Bailey and Morley, 2006; Holt et al., 2018), a better understanding of the empirical support for sport participation models emerges as a top research priority to address (Holt et al., 2018). Some sport participation and physical activity-based models could also be useful in understanding sport involvement in the general population. Sport participation and physical activity-based models characterize different stages of sport involvement based on sport activities (organized and unorganized) individuals take part in at different age markers (Côté et al., 2007; Balyi et al., 2013). These models also help explain how an individuals' sport participation can take shape over time by suggesting different pathways of sport participation. For example, these models suggest that during childhood, youth can either play multiple sports (e.g., sport sampling) or participate intensively in only one sport (e.g., sport specialization). Then, around adolescence, youth can either specialize into a single sport (e.g., elite participation) or maintain recreational involvement in physical activity (Côté et al., 2007; Balyi et al., 2013). Given estimates that nearly one third of youth drop out of sport annually during adolescence (Delorme et al., 2011; Fraser-Thomas et al., 2016), evidence-based sport participation and physical activity-based models can represent powerful tools for planning successful age-appropriate interventions to promote sport and physical activity participation. Sport participation and physical activity-based models contrast models aimed at explaining various factors associated with sport career or talent identification. Whereas sport participation and physical activity-based models attempt to describe patterns of sport or physical activity participation throughout various stages of life, other models seek to describe how various specific factors (e.g., psychological characteristics Abbott and Collins, 2004, participation environment Henriksen et al., 2010a, career transition Stambulova et al., 2009, motor skill acquisition Starkes et al., 2004) may facilitate or hinder talent development or identification, typically among elite athletes.

Although some reviews of the sport participation model literature have been undertaken, no review sought to investigate whether or not current models of sport participation are applicable to describe sport involvement in the general population. Past reviews have identified the historical development of athlete development models (Bruner et al., 2009), detailed interconnections among various sport participation models (Bruner et al., 2010), highlighted gaps in the literature to justify the development of a new model (Gulbin et al., 2013), and described the empirical support for a specific sport participation model (Côté and Vierimaa, 2014). Despite these efforts, criticism surrounding the empirical basis for the development of certain sport participation models exists (Bailey et al., 2010; Ford et al., 2011; Collins and Bailey, 2013) and some researchers have argued that underlying tenets of sport participation models are too restrictive to adequately represent the sport experience of most participants (Güllich, 2014, 2017; Güllich and Emrich, 2014; Cupples et al., 2018). For example, one model suggests that around age 16, sport participants have developed the physical, cognitive, social, emotional, and motor skills needed to invest their effort into highly specialized training in one sport (Côté et al., 2007). However, research among elite athletes suggests that identification of a specific age for specialization might not be appropriate since there are multiple pathways that can lead toward elite (e.g., national/world-level) success (Güllich, 2014; Huxley et al., 2017; Cupples et al., 2018). Further, it is unknown whether or not this is applicable to the general population where sport performance might not necessarily be a goal of sport participation. Therefore, it is unclear if sport participation and physical activity-based models can be applied to describe patterns of sport participation in the general population.

Uncertainty therefore remains regarding the empirical evidence of the foundational principles contained in participation and physical activity-based models (Holt et al., 2018). Identifying the breadth and depth of research surrounding these tenets is a tenable way of assessing the quantity and quality of the existing literature (Levac et al., 2010). Doing so will also provide direction for future research (Levac et al., 2010). To fill this gap, the current scoping review sought to map sport participation models and to investigate empirical support for the principles of sport participation and physical activity-based models aimed at describing the evolution of sport participation among individuals in the general population. Given the aims of the review were exploratory in nature (e.g., to inform on the current state of evidence and to provide direction for future research), a scoping review approach was selected as it lends itself to the objective of providing a broad, yet preliminary overview of the subject (Lockwood and Tricco, 2020).

## Methods

### Design

This scoping review is informed by the Preferred Reporting Items for Systematic Reviews and Meta-Analyses (Tricco et al., 2018) and the methodology outlined by Levac et al. (2010) for conducting scoping reviews. Two overarching phases were used: First, we identified models that relate to sport participation in the literature and retained those that described the evolution of an individual's sport participation over time (i.e., sport participation and physical activity-based model). Second, we identified the extent and nature of the evidence for the various tenets of the models identified.

### Phase 1: Identifying Sport Participation Models

#### Sport Participation and Physical Activity-Based Model Identification

Using the study selection approach suggested by Levac et al. (2010), a snowball methodology was employed to identify models that relate to sport participation. First, two published, peer-reviewed articles were used as a starting point for identifying sport participation models because they provided important insight into the historical development of sport participation models. We believed that these two articles would provide a practical initial list of sport participation models. The first article traced the origins of athlete development models in sport (Bruner et al., 2009) and identified five different models of sport participation. The second article consisted of a citation network analysis (Bruner et al., 2010) built upon the first article and added two additional models of sport participation. These seven sport participation models were used as a starting point for identifying additional models. Specifically, in the articles that first described each of these seven different models of sport participation, we searched the references for cited literature, book chapters, and peer-reviewed papers that described models of sport participation that were not initially identified. If additional models were cited, then these were read to identify if they described sport participation.

#### Sport Participation and Physical Activity-Based Model Classification

Literature describing each identified model was read so that they could first be classified according to the context in which the model is used. For this, models were categorized as (i) describing activities relating to sport participation (i.e., sport participation characteristics, skill development, sport environment characteristics, etc.), or (ii) applicable to, but not specific to sport development (i.e., models for education or general skills development). Second, the models were also classified based on their primary aims. Specifically, the models were classified as either describing talent development, talent identification or career transition. Talent development models refer to the process through which talent is developed to lead to elite performance or the processes that lead to recreational sport participation (Simonton, 1999; Gagné, 2004). Talent identification models aim to define the characteristics of individuals with potential to succeed in senior elite sport (Vaeyens et al., 2009; Johnston et al., 2018). Career transition models describe the strategies required to advance to a further development level (Stambulova et al., 2009).

#### Content Validation of Sport Participation and Physical Activity-Based Models

Levac et al. (2010) advocate for expert consultation, adding that “preliminary findings can be used as a foundation to inform the consultation.” To determine if the list of models identified in the first phase of the review was exhaustive, a two-step content validation process was used. First, the authors each read literature on each of the models identified and worked collaboratively to classify them. Second, we validated that the list was exhaustive with international experts in sport participation. Experts were first authors of the identified models or other authors commonly publishing on sport participation development. Each expert was e-mailed a two-page description of the background and aims of the review with a figure presenting models as classified using the logic described above. Experts were asked to comment on whether the list of models was exhaustive and to suggest modifications or additions. The experts were contacted by up to three e-mails each. We then reviewed any supplementary material recommended by the experts.

#### Selection and Description of the Sport Participation and Physical Activity-Based Models

Because of our interest in characterizing sport participation across the lifespan, we were specifically interested in models that can characterize the evolution of sport participation for a given individual. Therefore, talent development models were considered given their broad aim. Explanatory models were excluded since these models are aimed at explaining how specific factors [e.g., psychological characteristics (Abbott and Collins, 2004), participation environment (Henriksen et al., 2010a), career transition (Stambulova et al., 2009), motor skill acquisition (Starkes et al., 2004)] may facilitate or hinder talent development or identification, and do not necessarily encompass the entire lifespan. We excluded talent identification models since these are mostly concerned with identifying which athletes make the transition into elite levels of competition (Vaeyens et al., 2009; Johnston et al., 2018), and thus fail to describe the evolution of a participant throughout his/her lifespan. We also excluded career transition models since they focused on elite athletes transitioning out of sport, and therefore only encompass a small proportion of an athlete's life. Next, we excluded models that did not describe participation development within a general sport context since models developed in “other contexts” may not be readily applicable to sport. Models specific to one sport were also excluded since they focus on specific development elements in a given sport and they might not be applicable to other sports or general sport participation. Additionally, since many sport federations have their own model, we excluded models specific to a single sport for feasibility reasons. Finally, the tenets (underlying principles) of models retained were extracted and then used to provide a description of the sport participation models.

### Phase 2: Identifying Evidence for Sport Participation and Physical Activity-Based Models

#### Search Strategy and Data Sources

Based on the premise that scientific articles testing tenets of sport participation models will have cited manuscripts detailing the model of interest, we searched for manuscripts that cited the original articles presenting each of the models retained through Phase 1 of this study. Specifically, we identified literature specific to the sport participation models through the Scopus online database cited-by tool. This allowed to retrieve articles that cited one of the original articles presenting the models retained in phase 1. As of January 2020, the Scopus online database was comprised of five independent indices providing access to over 23,000 peer-reviewed journals, including 1.7 billion cited references since 1970 (Scopus, 2020). After identification in the Scopus online database, we used our institutional online library to collect articles. No publication date restrictions were used and the literature search was conducted between July 2019 and August 2020.

#### Eligibility Criteria

Original research articles investigating one or more of the tenets underlying the models met the inclusion criteria of the current review. Studies published in peer-reviewed journals in French or English were included for review.

#### Study Selection and Data Collection Process

After removal of duplicates, all titles and abstracts were scanned for the eligibility criteria. Full texts were then read to determine the tenet that was investigated. In cases where a study did not explicitly cite a tenet of the model under review, but tested something similar to a tenet, the article was read by the other co-author to determine if that specific study should be included or not in the current analysis. Any disagreement was resolved by discussion between co-authors.

Data from all included studies were recorded in one main data extraction sheet (Appendix). Extracted data included: (a) study characteristics (e.g., author names, year, and country of origin); (b) major focus of the study; (c) study design and measures; (d) sport(s) investigated and level of competition; (e) sample description; (f) main results; (g) tenets investigated.

## Results

### Identification of Sport Participation and Physical Activity-Based Models

#### Content Validation

The snowball strategy described above led to the identification of 16 models that relate to sport participation. A total of 16 experts were then identified and invited to validate the content of this list. Four e-mails were invalid. Of the 12 experts remaining, three did not answer and one responded that they did not consider themselves an expert on the topic and was thus excluded from the expert count, for a response proportion of 72% (8/11). Most experts confirmed that the list of identified models was exhaustive, but others suggested additional readings on supplementary models, including books, chapters, and selected articles. This resulted in the inclusion of one additional model for consideration in the review (total of 17 models). The other recommended models did not fit the criteria for the current review; some documents described models that are specific to a single sport, while others did not fit into the scope of the current study (i.e., positive youth development).

#### Model Selection

**Figure 1:**
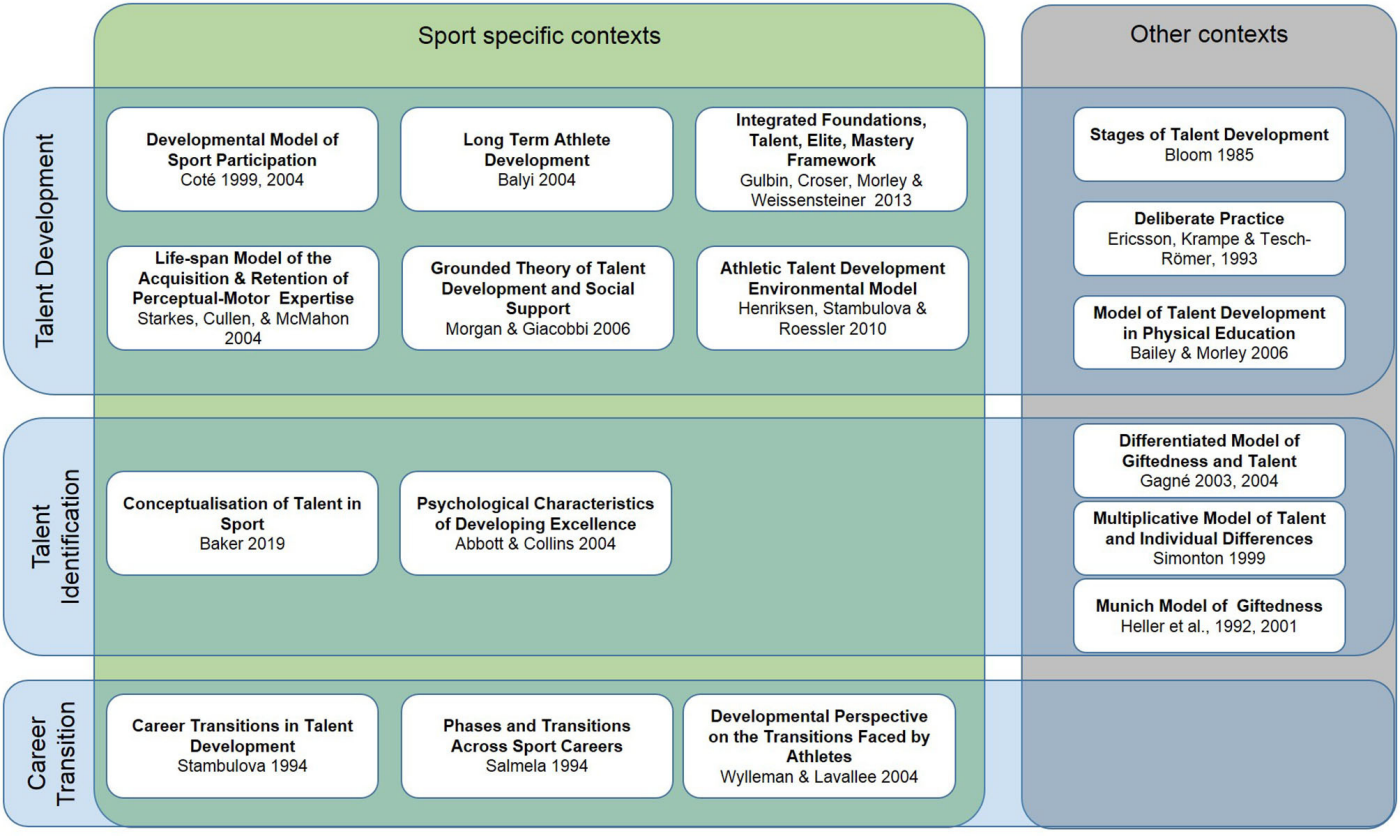
Sport participation development models in sport specific and other contexts.

Of the 17 models considered, 11 offered a description of individuals' development in a sport context specifically, whereas six could be applied to other contexts. Among the 11 models specific to the sport context, six described talent development, two described talent identification and three described career transitions as their primary aims (Figure 1).

Of the six models that describe talent development in sport, three models can be considered sport participation and physical activity-based. They characterize sport participation throughout the lifespan by describing sport and activity involvement and were retained for this review: the Developmental Model of Sport Participation (Côté, 1999; Côté et al., 2007) (DMSP), the Long-Term Athlete Development (LTAD) Model (Balyi et al., 2013), and the Integrated Foundations, Talent, Elite, Mastery (FTEM) framework (Gulbin et al., 2013). The three models not retained provide important additions to the literature, but focus on specific aspects of sport participation and do not necessarily describe the activities participants take part in of across the lifespan. Specifically, the Life-span Model of the Acquisition & Retention of Perceptual-Motor Expertise was excluded since it explains how individuals acquire and retain motor skills, but does not characterize sport participation (Starkes et al., 2004). Similarly, the Grounded Theory of Talent Development and Social Support model explains the importance of social support in the development of highly successful collegiate athletes, but does not characterize sport participation (Morgan and Giacobbi, 2006). Finally, the Athletic Talent Development Environmental Model describes the environmental characteristics that might favor successful transitions from junior elite status to senior elite status (Henriksen et al., 2010a). It was excluded since it is interested in environmental characteristics and not activities that characterize sport involvement.

The three models retained are stage-based, meaning that they describe development of sport participation as going through successive stages. All models suggest appropriate sport activities for their various stages, but differ in their definition of boundaries. For example, stages in the DMSP are based on participants' age, while stages in the LTAD are based on participants' age and biological development. The FTEM framework stages are delimited by sport competency or level of competition and allows for non-linear movement between stages.

All three models advocate for participation in multiple sports during childhood, but also recognize that some participants and/or some sports require early specialization. In general, tenets of the three models address youth sport participation practices (i.e., sport sampling vs. early sport specialization) and long-term physical activity involvement, describe the adequate type of sport involvement at a given stage (i.e., deliberate practice, deliberate play, development of physical literacy), or psychological characteristics required to successfully navigate through the various stages of each model. A detailed description of each tenet of the three sport participation and physical activity-based models is available in Table 1.

**Table 1:** Tenets of the sport participation and physical activity-based models retained and references supporting them.

**Tenets of the Developmental Model of Sport Participation (DMSP)**	**References**
**Tenet #1**: early diversification (sampling) does not hinder elite sport participation in sports where peak performance is reached after maturation.	Baker et al., 2003; Barreiros et al., 2013; Coutinho et al., 2014, 2016; Güllich and Emrich, 2014; Güllich, 2017; Huxley et al., 2017; Cupples et al., 2018
**Tenet #2**: early diversification (sampling) is linked to a longer sport career and has positive implications for long-term sport involvement.	Baker et al., 2005; Fraser-Thomas et al., 2008; Bridge and Toms, 2013; Coutinho et al., 2014, 2016; Gallant et al., 2017
**Tenet #3**: early diversification (sampling) allows participation in a range of contexts that most favorably affects positive youth development.	
**Tenet #4**: high amounts of deliberate play during the sampling years build a solid foundation of intrinsic motivation through involvement in activities that are enjoyable and promote intrinsic regulation.	Hendry et al., 2014, 2019a; Vink et al., 2015; Forsman et al., 2016; Thomas and Güllich, 2019
**Tenet #5**: a high amount of deliberate play during the sampling years establishes a range of motor and cognitive experiences that children can ultimately bring to their principal sport of interest.	Baker et al., 2012; Haugaasen et al., 2014; Güllich, 2017, 2018; Sieghartsleitner et al., 2018
**Tenet #6:** around the end of primary school (about age 13), children should have the opportunity to either choose to specialize in their favorite sport or to continue in sport at a recreational level.	Soberlak et al., 2003; Hayman et al., 2011; Coutinho et al., 2014, 2016; McFadden et al., 2016
**Tenet #7**: late adolescents (around age 16) have developed the physical, cognitive, social, emotional, and motor skills needed to invest their effort into highly specialized training in one sport.	Moesch et al., 2011; Ginsburg et al., 2014; Güllich, 2014; Güllich and Emrich, 2014; Hornig et al., 2016; Huxley et al., 2017, 2018; Mendes et al., 2018; Hendry et al., 2019b
**Tenets of the Long-term Athlete Development Model**	**References**
**Physical Literacy**: Physical literacy is the development of a range of basic human movements, fundamental movement skills, and foundational sport skills that give people the tools to engage in health-enhancing physical activity for life—to be active for life. Required for movement through the sport excellence stages.	
**Specialization**: When children try a number of sports and choose to specialize later, they increase their chances of excelling (movement patterns, decision making). Some sports require early involvement. Some sports require early specialization.	Arede et al., 2019; Yustres et al., 2019
**Age** (Chronological, skeletal, relative, developmental, general training, sport-specific training): Chronological age is not a good predictor of developmental age. Monitoring relative age is essential, because those born early in the active year have initial advantages and those born late have initial disadvantages. Identifying early, average, and late maturers during puberty is essential for providing developmentally appropriate training, competition, and recovery programs.	McCunn et al., 2017
**Trainability**: Windows of opportunity exist for accelerated development of the 5S's (Strength, Speed, Skills, Stamina, Suppleness) around puberty based on specific biomarkers (e.g., PHV).	Moran et al., 2018
**Intellectual, emotional, moral development**: When we consider athletes' readiness, we take into account physical, intellectual, emotional, and moral development. Athletes develop in these areas at varying speeds, which can affect their capacity to deal with the overall sport experience. Their level of capacity determines when to move from one stage to the next. Because of this, athlete development should be individualized.	
**Excellence takes time**: To achieve expertise in an activity, people require thousands of hours of practice over the span of approximately a decade (depending on activity, coaching, natural ability). Unstructured and free play in other activities are beneficial. By developing a variety of skills over a range of sports and activities, athletes are often better equipped to excel in a single sport later on. Premature selection deprives some youth of the chance to pursue the thousands of hours they need to achieve excellence. Requires appropriate support (environment).	
**Periodization**: Training must be planned appropriately.	
**Competition**: Many facets of competition must be considered and modified if children are to develop properly and want to remain in the game. As competition should be modified to suit children and youth, care must also be taken to avoid overcompetition.	
**System alignment and integration**: LTAD cannot be fully achieved without the health, recreation, sport, and education sectors working in tandem. System alignment leads to increased quality in sport and physical activity, which encourages more participation, which in turn creates more sustainable activity.	Kristiansen et al., 2018
**Continuous improvement**: Coaches need to worry less about strategizing with young players and more about the overall development of each athlete. Rules encouraging less-result-oriented approaches should be incorporated.	
**Tenets of the Foundations, Talent, Elite, Mastery (FTEM) framework**	**References**
**(F1) Learning and acquisition of basic movement foundations:** Participant's early exposure to a variety of movement experiences that afform them a broad range of essential movement foundations.	
**(F2) Extension and refinement of movement foundations**: Advance and refine F1 movement experiences through continued broad exposure to formal and informal play, practice and games, in both sport specific and non-sport specific ways (i.e., sport sampling).	
**(F3) Sports specific commitment and/or competition:** Increase in the commitment to training, sport specific skill development, and/or formal engagement in competition. Can also include the pursuit of personal improvement or self-competition, through either a competitive or non-competitive environment.	
**(T1) Demonstration of high performance potential**: Athletes typically exhibit demonstrable and measurable gifts or talents in one or more of the physical, physiological, psychological, and skill domains, which indicate future potential in high performance sport.	
**(T2) Talent verification:** Evidence based observations (T1) should be supplemented by the subjective judgements and intuition of experienced coaches.	
**(T3) Practicing and achieving**: Having gained interest from talent scouts, coaches or national sporting organizations, athletes are committed to higher levels of sport specific practice and striving for continual performance improvements that are focused on a benchmark outcome.	
**(T4) Breakthrough and reward**: Gaining professional support for continued development (athletic scholarship at University or institute or academy of sport, or are drafted into a professional team or elite training squad.	
**(E1) Senior elite representation:** Olympic/World: national team selection; Professional: playing at highest level of professional competition.	
**(E2) Senior elite success:** Olympic/World: podium finish; Professional: professionally successful (established metrics/accolades in their sport).	
**(M) Mastery:** Olympic/World: podium in two games (8 years); Professional: success over several seasons/period/era.	

### Empirical Evidence for Sport Participation and Activity-Based Models

#### Study Characteristics

**Figure 2:**
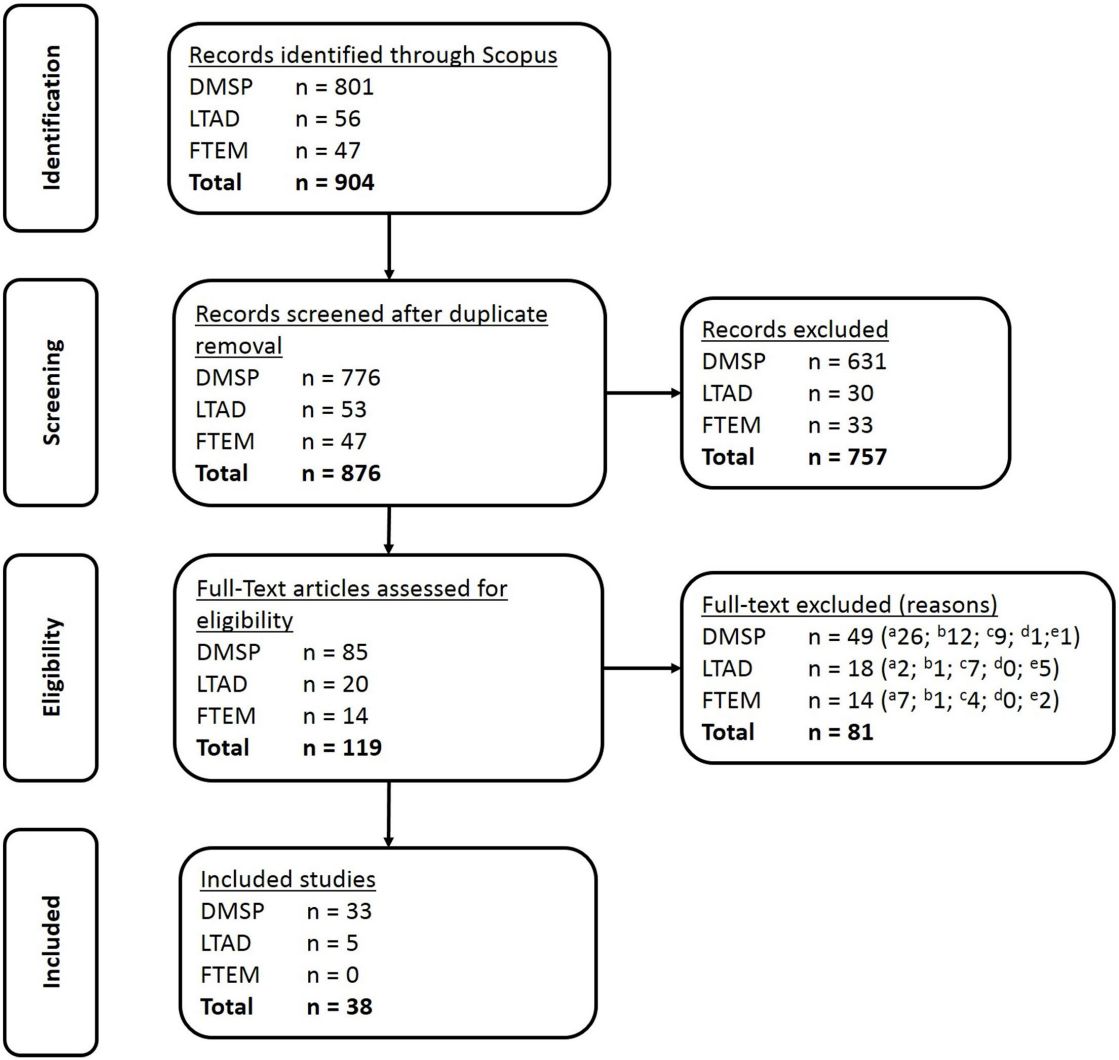
PRISMA flow diagram of study selection process. DMSP, Developmental Model of Sport Participation; LTAD, Long Term Athlete Development Model; FTEM, Foundation, Talent, Elite, Mastery Framework Reasons for full-text exclusion. ^*a*^only mentions model/no testing of tenets; ^*b*^objective of paper is talent identifiaction or career transition; ^*c*^review paper; ^*d*^does not talk about sport; ^*e*^focused on coaching/sport governance rather than sport participation development.

After removal of duplicates, a total of 876 articles were identified as citing one of the three models. Based on titles and abstracts suggesting assessment of a tenet, 119 articles were retained and screened. Of these, 38 articles were considered to test one or more of the tenets of either the DMSP, the LTAD or the FTEM and were included in the review (Figure 2). The most common reason for excluding articles was that they only mentioned a model, but did not test tenets of a model. Other common reasons for excluding manuscripts were that the objectives were related to talent identification (e.g., describe the skills and/or physiological/psychological characteristics of selected/unselected athletes) or career transition, or that they were review articles. We extracted data from these articles which allow to summarize the model under study, methodologies undertaken, variables assessed and sample sizes included (Table 2).

**Table 2:** Summary of study characteristics providing assessment of sport participation and physical activity-based models' tenets.

**Model** **(*n* studies)**	**Country** **(*n* studies)**	**Design** **(*n* studies)**	**Main outcome measures** **(*n* studies)**	**Sample size** **(*n* studies)**
Developmental Model of Sport Participation (33)	Canada (9) Germany (8) UK (4) Portugal (3) Australia (3) Finland USA Brazil Denmark Switzerland Estonia	Cross-sectional (24) Prospective/cohort (4) Comparative (2) Qualitative (1) Matched-pairs design (1) Mixed-methods (1)	Structured retrospective interviews (32) Sport Motivation Scale (2) Behavioral Regulation in Sport Questionnaire (2) Psychological Skills Inventory for Sports (1) DMSP-based categorizations based on self-reported organized and unorganized physical activity (1)	≤ 20 (3) 21–50 (8) 51–100 (7) 101–250 (9) 251–500 (3) >500 (3)
Long-Term Athlete Development model (5)	Portugal Norway Germany UK Spain	Retrospective (3) Quasi-experimental (1) Repeated cross-sectional (1)	Test performance (2) Still participating in sport (1) Selection to team (1) Sport performance (1)	21–50 (1) 51–100 (2) 251–500 (1) >500 (1)

Of all the models considered for the review, the DMSP received the most support (*n* = 33/38), the LTAD received support from five articles, while the FTEM framework did not receive any support. Support for the models came from 13 different countries, with most publications from Canada (*n* = 11), Germany (*n* = 9), and the United Kingdom (*n* = 7). Seventy-one percent (*n* = 27/38) of the studies used a cross-sectional design. Twenty-one studies had sample sizes ≤ 100, nine studies had sample sizes between 101 and 250 participants, and eight studies had sample sizes ≥ 500.

#### Support for the Sport Participation and Physical Activity-Based Models

The empirical support identified for each individual tenet of the sport participation and physical activity-based models is summarized in Table 1. Nearly all studies investigated differences in career development (i.e., hours of training, age of attaining milestones, level of performance) between elite and non-elite athletes and employed a retrospective interview protocol as a primary measure (*n* = 34/38). While there were differences between studies for their interview protocols, most used retrospective questions for information regarding demographics, early activities (i.e., timeline of involvement in all structured leisure activities throughout development, including number and time spent in each), developmental milestones (i.e., age at which the athlete reached key sport-related milestones), and sport-specific activities (i.e., time spent in specific training activities at each stage of development). The remainder of the studies investigated differences between elites and non-elites regarding performance on physical tests or psychological characteristics. Only one study prospectively investigated a sample in the general population that was classified into different profiles of sport participation based on their longitudinal self-reported level of involvement in organized and unorganized physical activity during adolescence (Gallant et al., 2017).

##### Support for the DMSP

Tenet #1 of the DMSP suggests that sampling many sports early in the sporting career does not hinder elite sport participation in sports where peak performance is reached after maturation. This was the most commonly assessed tenet identified in this review. In this context, all studies used a retrospective questionnaire to compare developmental differences between elite (i.e., senior international competition or professional contracts) and non-elite (i.e., junior international level or below) developmental pathways of athletes (Baker et al., 2003; Barreiros et al., 2013; Coutinho et al., 2014, 2016; Güllich and Emrich, 2014; Güllich, 2017; Huxley et al., 2017; Cupples et al., 2018). All studies supported the notion that taking part in many sports during the developmental years does not prevent against becoming an elite athlete. For example, in a sample of professional rugby players, the number of other sports athletes took part in during their developmental years was undistinguishable between athletes who played on an elite-level team early (U-16 or U-18 level) or later (U-20 level) (Cupples et al., 2018). Similar results were observed in studies comparing elite and non-elite volleyball players (Coutinho et al., 2014, 2016), and triathletes (Baker et al., 2005).

Tenet #2 of the DMSP suggests that early sampling is linked to longer sport involvement. Of the studies supporting this tenet, five are cross-sectional (Baker et al., 2005; Fraser-Thomas et al., 2008; Bridge and Toms, 2013; Coutinho et al., 2014, 2016) and one is prospective (Gallant et al., 2017). For example, in a cross-sectional study among 18 year old non-elite swimmers, participants who dropped out more often had an early sport participation profile characterized by early specialization (Fraser-Thomas et al., 2008). One study investigating the natural history of youth participation also demonstrated that sampling many sports at the end of childhood was positively associated with an increased probability of still participating in sport at 15 years old, while specializing in one sport at the end of childhood did not protect against dropout (Gallant et al., 2017). Similarly, another study found that compared to taking part in one sport, individuals taking part in at least three sports at 11, 13, and 15 years had a higher likelihood of playing at a higher competitive level at 16 and 18 years (Bridge and Toms, 2013). Finally, while the age range of studies supporting this tenet is limited, it does support that participating in multiple activities during childhood helps maintain sport involvement, in some cases, until at least 30 years.

Tenet #3 of the DMSP states that early sport sampling favorably affects positive youth development. Positive youth development is a framework aimed at developing competence, confidence, connection, character, and compassion/caring among youth (Shek et al., 2019). The methodology used to retrieve articles that cited and tested tenets of the sport participation and physical activity-based models in this review did not lead to identification of articles assessing this tenet.

Tenet #4 of the DMSP suggests that deliberate play during the sampling years helps build intrinsic motivation toward the practiced sport. While no study investigated deliberate play on intrinsic motivation specifically, four studies investigated the relationship of both play and practice with motivation (Hendry et al., 2014, 2019a; Vink et al., 2015; Thomas and Güllich, 2019) and one investigated the link between practice only and motivation (Forsman et al., 2016). Supporting this tenet, a study of Finnish athletes found a positive relationship between total accumulated sport-specific play and practice and motivation (Forsman et al., 2016). Similarly, one longitudinal study demonstrated that taking part in more deliberate practice among U-15 elite athletes was associated with higher intrinsic motivation 12 months later (Vink et al., 2015). In contrast, another study found a negative relationship between among of practices during childhood and autonomous motivation among teenaged soccer players (Hendry et al., 2019a). Two other cross-sectional studies found no statistically significant association between the amount of play and intrinsic motivation (Hendry et al., 2014; Thomas and Güllich, 2019). The results from these studies are therefore mixed and do not clearly demonstrate that deliberate play is positively associated with intrinsic motivation.

Tenet #5 of the DMSP suggests that deliberate play during the sampling years exposes youth to motor and cognitive experiences that can positively influence a transfer of skills to the principal sport of interest. While no studies have demonstrated that only deliberate play positively influences skill transfer, the studies that tested this tenet investigated sport involvement in general (i.e., play and practice) and speculated that sampling many sports early positively influenced later sport performance (Baker et al., 2012; Haugaasen et al., 2014; Güllich et al., 2017; Güllich, 2018; Sieghartsleitner et al., 2018). In one longitudinal study of elite U-12 soccer players, participating in more non-organized soccer play and practice/training in other sports was positively associated with greater improvements in soccer match play two-years after baseline (Güllich et al., 2017). In one study of track and field athletes comparing participants on their level of improvement in performance over time, participants who showed greater improvements reported taking part in more sports over more years, compared to those who did not improve as much before specializing in athletics (Güllich, 2018). These results suggest that exposure to many sport experiences through sampling during youth helps future sport performance; specifically, a combination of play and practice might be necessary to facilitate transfer of skill between sports.

Although not as frequently assessed as tenet #1, tenets #6 and #7 were the subject of a relatively large number of scientific articles. Tenet #6 suggests that around age 13, children should have the opportunity to either specialize in their favorite sport or continue participating in sport at a recreational level, whereas tenet #7 of the DMSP suggests that athletes should only dedicate themselves to a single sport from around the age of 16. These tenets are in line with many studies supporting tenets #1 (elite participation following sampling) (Baker et al., 2003; Barreiros et al., 2013; Güllich, 2017) and #2 (sampling leads to long-term involvement in sport) (Baker et al., 2005; Coutinho et al., 2014, 2016) regarding typical sampling-specializing-investment transitions to achieve elite status. However, multiple studies investigating appropriate timing for specialization inconsistently provide specific ages for professional (Hayman et al., 2011; Ginsburg et al., 2014; Hornig et al., 2016; Cupples et al., 2018; Güllich, 2019), and world or Olympic caliber athletes (Moesch et al., 2011; Huxley et al., 2018; Mendes et al., 2018). While some studies demonstrate timing in concordance with the suggested age ranges (Soberlak et al., 2003; Hayman et al., 2011; Coutinho et al., 2014, 2016; McFadden et al., 2016), most studies demonstrate that many successful athletes only specialized after 16 years (Moesch et al., 2011; Güllich, 2014; Güllich and Emrich, 2014; Huxley et al., 2017, 2018; Hendry et al., 2019b). Given the state of the current literature, typical sampling-specializing-investment transitions seem to be beneficial for attainment of elite-level sport participation, but specific age ranges have yet to be consistently demonstrated.

##### Support for the LTAD Model

Four of the ten tenets of the LTAD were supported by five articles. Two studies supported the specialization tenet (Arede et al., 2019; Yustres et al., 2019), which states that sampling many sports early increases the chances of excelling in sport and that different sports require different degrees and timing of specialization (Balyi et al., 2013). One study found that, compared to more specialized athletes, less-specialized athletes at the U-13 level had greater physical skills and had a greater chance of getting selected at the U-14 level (Arede et al., 2019). The other study conducted a secondary analysis of junior and senior world championship swimming performance and found a positive association between participation at the junior world championship and ranking at the senior world championship level (Yustres et al., 2019). The authors conclude that early specialization in swimming helps achieve senior success, but athlete training histories were not recorded and the junior world championships include a wide age range (between 13 and 18 years).

The tenet regarding age, suggesting that maturation rates should be consistently monitored to ensure developmentally appropriate training (Balyi et al., 2013), received support from one study. Specifically, a repeated cross-sectional study identified that when comparing elite youth soccer players born within the same calendar year, the relatively older players in the U-14 and U-15 age groups were more physically mature than same-age peers born later in the year and thus had greater sprint speed (McCunn et al., 2017). This finding supports the tenet in that those born early in the active year might have initial advantages than those born later, indicating a need for continuous monitoring of the developmental age of athletes.

One study was found to assess the tenet of trainability, which states that windows of opportunity exist for accelerated development of certain abilities (i.e., speed) (Balyi et al., 2013). This study demonstrated that athletes who were developmentally younger based on age at peak height velocity, a marker of pubertal development, responded better to sprint training than athletes who were in their growth spurt (Moran et al., 2018). This finding suggests that physiological responses to training might differ according to individual developmental patterns.

Finally, the tenet stating that the LTAD cannot be fully achieved without alignment of various sectors (i.e., health, recreation, sport, education) (Balyi et al., 2013) was also tested in one study. Specifically, using an internet-based survey of 58 athletes who participated in the Youth Olympic Games, 29% had dropped out of sport thereafter. One of the most frequently reported reasons of drop out among these young athletes whom had had success on the world stage related to the difficulties managing education and training (Kristiansen et al., 2018). These results support the tenet of alignment, whereby multiple sectors must interact to successfully support an athlete throughout development.

## Discussion

This review summarized the current state of evidence in support of sport participation and physical activity-based models aimed at characterizing the evolution of sport participation by describing sport activity involvement over time. In the wider sport model literature, a total of 17 models were identified and classified according to their context of use and their main aim. A total of three models offered a description of the activities participants take part in throughout their lifespan (Côté et al., 2007; Balyi et al., 2013; Gulbin et al., 2013). Empirical support for these three models varied. Consistent with past research (Bruner et al., 2010; Côté and Vierimaa, 2014), the DMSP is the most empirically supported sport participation and physical activity-based model, while the LTAD received some support and the FTEM framework received no support. Further, some tenets of the models were assessed and supported, while some were not tested or received no support. Overall, cross-sectional studies of elite athletes that used retrospective data collection methods was the most popular type of study used to assess tenets of the sport participation and physical activity-based models.

### Long-Term Sport Participation

The three models describing the evolution of sport participation suggest a life-long sport participation pathway (i.e., for non-elite athletes/general population) (Côté et al., 2007; Balyi et al., 2013; Gulbin et al., 2013). All three models also advocate for sport sampling as an effective way of increasing the chance of sport participation over the lifespan. However, the current review found limited support for associations between sampling and long-term sport participation in the general population. For example, only one study in the current review investigated longitudinal associations between sport sampling and sport specialization in the general population. In the study, sport sampling at age 10 protected against sport dropout in the following five years, but early sport specialization did not (Gallant et al., 2017). While this study provides some support for the benefits of sport sampling during childhood, the narrow age range of participants does not inform on sport participation development beyond adolescence. Nevertheless, other longitudinal studies conducted in the general population corroborate the benefits of playing multiple sports. Russell and Limle (2013) demonstrated that physically active college students were more likely to have played more sports during their teens than physically inactive college students. Likewise, participating in different types of sports during adolescence was positively associated with the physical activity levels of Finnish adults, but the number of sports played during adolescence was only positively associated with physical activity levels of Finnish women (Mäkelä et al., 2017). Recognizing that individuals participate in sport differently, an update to the LTAD model has seen the inclusion of “competitive for life” and “active for life” pathways (Higgs et al., 2019) (such pathways are already identified in the FTEM Gulbin et al., 2013). Given the importance of sport and physical activity participation throughout the lifespan (Warburton et al., 2006; Nelson et al., 2007; Janssen et al., 2010), more studies need to investigate the natural development of sport participation among the general population and to identify predictors and outcomes of sport participation across the lifespan. This information will be crucial to improve our understanding of general patterns of sport participation and will help refine recreational sport participation pathways described in sport participation and physical activity-based models currently used.

### Non-linearity of Sport Participation

All three reviewed models are stage-based (i.e., sport participants must demonstrate certain abilities or characteristics before transferring from one stage to the next). While the DMSP and the LTAD both describe participation development as linear pathways, the FTEM framework does not refer to linear transitions between stages (Gulbin et al., 2013). Some research included in this review suggests that the linearity of transitions described by the DMSP and LTAD might not reflect actual sport participation pathways of most participants. For example, several different trajectories were found to be associated with eventual professional rugby (Cupples et al., 2018) or golf (Hayman et al., 2011) contracts, Olympic appearances in track and field (Huxley et al., 2017), and other elite athletic achievements (Storm et al., 2012). Longitudinal studies spanning childhood to early adulthood have also demonstrated that several different trajectories of sport participation exist in the general population (Rodriguez and Audrain-McGovern, 2004; Findlay et al., 2009; Kwon et al., 2015; Howie et al., 2016). Therefore, more longitudinal research identifying the various paths sport participation may take are needed to perfect sport participation pathways described in models.

Sport participation and activity-based models should give increased attention to dropout in sport, since most participants will discontinue sport participation at some point (Butcher et al., 2002; Fraser-Thomas et al., 2016). For example, a study conducted among 1,300 tenth graders identified that 94% of participants had dropped out of at least one sport since grade 1, but that more than half of the participants who dropped out in grade 7 or 8 had taken up a new sport thereafter (Butcher et al., 2002). Further, the study identified that dropout can be activity-specific (discontinuing a specific activity) or domain-general (withdrawal from all sport activities) (Butcher et al., 2002; Fraser-Thomas et al., 2016). These findings highlight that sport participation during youth is especially volatile and that over-and-above simply identifying dropout as a possible outcome of sport participation, an increased discussion and awareness around sport dropout and uptake could improve sport participation models. Given that different activities have different dropout rates over time (Butcher et al., 2002; Bélanger et al., 2009), and that activity-specific dropout is central to sport participation profiles (i.e., sport sampling; sport specialization), not considering the difference between activity-specific or domain-general dropout could further confuse our understanding of how youth sport participation evolves over time.

### Need to Improve Terms and Concepts

The models included herein each contained specific underlying tenets describing sport development. Several tenets were nevertheless similar across models. Yet, tenets from different models are difficult to compare since their terms and concepts are generally defined ambiguously (Güllich, personal communication, 2019). For example, in the absence of a standardized definition of “sport specialization,” at least five different definitions of this term can be found in the literature (DiSanti and Erickson, 2019). Similarly, there is no accepted operationalization of “sport sampling” or multi-sport participation. This ambiguity hampers empirical testing and model comparison, though some authors have made attempts at objectively operationalizing these concepts (Jayanthi et al., 2015; Gallant et al., 2017; Desroches et al., 2019). Further, consistent with past research (Swann et al., 2015) the current review found no cohesion among studies regarding the definition of “elite,” as some articles used “elite” to describe athletes who represent their country in international senior competitions (i.e., Olympics or World Championships) (Moesch et al., 2011; Güllich and Emrich, 2014; Güllich, 2017; Huxley et al., 2018), whereas others, used the term in reference to youth affiliated with a professional sport school (Güllich et al., 2017; Sieghartsleitner et al., 2018; Hendry et al., 2019a).

Notwithstanding the ambiguity among the terms and concepts, the homogeneity of research methodologies in the included articles is also noteworthy. The vast majority of included studies used cross-sectional research designs and investigated developmental histories of athletes using retrospective methods, echoing findings of a recent scoping review on sport specialization (DiSanti and Erickson, 2019). This suggests that the body of evidence is faced with the potential of important recall biases and threats to external validity. While retrospective interview protocols have demonstrated certain reliability parameters (Baker et al., 2003, 2005; Güllich, 2014; Güllich and Emrich, 2014), some athletes were asked to describe their training histories of at least two decades prior (Baker et al., 2003, 2005; Güllich, 2014). The longitudinal description of pathways is one strength of the models (Strachan et al., 2009), but methodologies undertaken to assess these fail to fully characterize participant development (DiSanti and Erickson, 2019). Further, cross-sectional research is limited in its' ability to assess causality. Since sport participation and physical activity-based models suggest that sampling may lead to elite sport participation, more advanced methods are needed to answer the fundamental question of whether elite athletes attained their status because they played multiple sports, or if they played multiple sports because they excelled in them (or had an affinity for sport in general).

### Limitations and Future Directions

Some limitations should be considered when reading the current review, including the possibility that it is not comprehensive since the databases used may not include all important peer-reviewed journals in the sport sciences field. Further, only articles that cited a model or specifically tested a tenet were retained for the current review. This ignores articles that have investigated associations supporting tenets of the sport participation models, but did not aim to do so for a model specifically. For example, our search strategy did not retrieve any articles aiming to test tenet #3 of the DMSP, which states that early sport sampling favorably affects positive youth development. However, there is consistent evidence that playing multiple sports is associated with better competence, confidence, and caring (i.e., markers of positive youth development) (Denault and Poulin, 2009; Agans and Geldhof, 2012; Agans et al., 2017). It is also important to acknowledge that models included herein were developed using existing evidence (Côté et al., 2007; Balyi et al., 2013; Gulbin et al., 2013), suggesting that from their inception they were already, at least partly, evidence-based. Whereas the goal of this review was to provide an overview of sport participation and physical activity-based models, including identification, classification and documentation of their empirical support, further reviews could assess detailed evidence for each of the tenets individually. Also, other models of sport development were identified but were not included in this review because they did not fit into the primary aim of the review, which was to investigate support for models that describe potential patterns of sport and physical activity participation throughout the lifespan (e.g., sport participation and physical activity-based models). For example, the Athletic Talent Development Environment model provides an important conceptualization of how the environment shapes elite athletes (Henriksen et al., 2010a,b), but does not describe the evolution of the participant throughout the lifespan. Similarly, the Abbott and Collins (2004) model provides an insightful discussion on the importance of considering psychological characteristics in talent development and identification programs, but is more focused on talent identification than talent development (Abbott and Collins, 2004). Although models describing the sport participation pyramid (Green, 2005; Bailey and Collins, 2013) were not included in this review since they were considered too broad to be able to characterize patterns of sport and physical activity participation, discussions surrounding access and opportunity for sport participation typically accompanying these models are worthwhile and should be considered when aiming to improve sport participation and physical activity-based models. Finally, in addition, models specific to any sport federations were not included for feasibility reasons as there are nearly as many models as there are sport federations.

The review has identified gaps in the current sport participation literature, which could be addressed in future work. In particular, this research has highlighted that there is little longitudinal research performed to support, or refute, tenets of sport participation and activity-based models. Therefore, future longitudinal investigations should aim to characterize a variety of patterns of sport participation in youth from the general population across multiple time points. In addition, given the paucity of identified research citing sport participation and physical activity-based models using samples of non-elite athletes (e.g., general population), it may be possible that not all sport and physical activity researchers know such models exist. Although knowledge users have identified athlete development models as a research priority (Holt et al., 2018), the extent to which researchers in broader physical activity and sport research are familiar with sport participation and physical activity-based models is unknown. Finally, efforts to standardize and operationalize terms and concepts included in sport participation and activity-based models could aid in providing evidence for all tenets proposed by such models.

## Conclusion

Although models identified herein are partly evidence-based, not all aspects of the models are empirically supported. Evidence for the tenets assessed generally originated from studies including considerable limitations and some tenets still need to be tested. Further, while models reviewed include a pathway for non-elite sport participation development, the research providing empirical support for models have largely focused on elite athlete development. More research and better description of non-elite sport participation pathways are therefore needed as they represent the trajectory of an important portion of sport participants. Efforts to clarify and describe terms and concepts used by the models are also needed to facilitate their conceptualization and to enable comparison across studies. Overall, the research reviewed in the current article suggests that some evidence support current sport participation and physical activity-based models, but that more research is required for them to be used in full confidence to guide sports policies, programs and practices, especially in the general population.

## Author Contributions

FG conceived the current study, performed the literature search, and drafted the initial manuscript. Both authors interpreted the data, revised the work critically for important intellectual content, and approved the final version to be published.

## Funding

FG was supported by a NBHRF & CIHR SPOR-MSSU Doctoral Studentship.

## Conflict of Interest

The authors declare that the research was conducted in the absence of any commercial or financial relationships that could be construed as a potential conflict of interest.

## Publisher's Note

All claims expressed in this article are solely those of the authors and do not necessarily represent those of their affiliated organizations, or those of the publisher, the editors and the reviewers. Any product that may be evaluated in this article, or claim that may be made by its manufacturer, is not guaranteed or endorsed by the publisher.
