# Supplementary material for: Empirical Support for the Tenets of Sport Participation and Physical Activity-Based Models: A Scoping Review
Source: Front Sports Act Living. 2021 Oct 14;3:741495. doi: 10.3389/fspor.2021.741495 (PMC8552970; doi:10.3389/fspor.2021.741495)
Supplement: Supplementary file 1 [file Table_1.docx]

| Appendix. Extracted data from the studies that supported tenets of the Developmental Model of Sport Participation | | | | | |
| --- | --- | --- | --- | --- | --- |
| Authors;  Year;  Country | Major focus of the study | Study design;  Measures | n (male:female)  **Level of competition** (description; age)  Sport(s) | Main findings | Tenet(s) tested |
| Baker, Bagats, Büsch, Strauss, Schorer;  2012;  Canada* Data from Germany | Investigate differences in accumulated training between young athletes being considered for selection to the next stage of skill development | Cross-sectional  Retrospective sport participation history | 129 (43:86)  **Elite** (selected for next stage of skill development; *M_Male_*=15.95, *SD_Male_*=0.21; *M*_Female_=14.91, *SD*_Female_=0.29)  **Near elite** (not selected for next stage of skill development; *M_Male_*=15.95, *SD_Male_*=0.21; *M*_Female_=14.91, *SD*_Female_=0.29)  Handball; | Selected females had accumulated more involvement in higher quality training than non-selected females. This was not the case in males. | Selected females accumulated more hours in more sports during early development that their peers (**tenet #5**) |
| Baker, Coté, Abernethy;  2003;  Canada*  (Australian athletes) | Examine the quantity and type of sport-specific and non-sport-specific practice accumulated of experts and non-experts | Cross-sectional;  Retrospective sport participation history | 28 (15:13)  **Expert** (national team caliber: all top 4 world; *M*=27.6, *SD*=4.3)  **Non-expert** (all minimum 10 years experience, did not participate beyond provincial level; *M*=23.2, *SD*=4.6)  Netball, Field hockey, Basketball | Experts trained more than non-experts during investment years. Experts decreased number of other sports near 12 years, while non-experts maintained involvement in multiple sports | Early specialization may not be a necessary requirement for exert level performance in decision-making sports (**tenet # 1**) |
| Baker, Côté, Deakin;  2005;  Canada | Examine the quantity and type of training performed by expert ultra-endurance athletes | Cross-sectional;  Retrospective sport participation history | 27 (27:0)  **Expert** (two *SD* above mean finishing time for their population=9h21; *M*=31.3, *SD* = 4.3)  ***M*id-pack** (mean finishing time=12h; *M*=32.3, *SD*=3.4)  **Back-pack** (slower than 2*SD*=15h; *M*=33.8, *SD*=4.4)  Triathlon | Number of sports did not distinguish between experts and non-experts, but experts performed more training than non-experts | While these findings support a diversified involvement in sport during early involvement (tenet #2), they also indicate that participation in other sports does not distinguish Expert and non-expert triathletes (**tenet #1**) |
| Barrerios, Coté, Fonseca;  2013;  Portugal | Explore activities, training patterns, and psychosocial influences of expert and non-expert athletes | Cross-sectional;  Retrospective sport participation history, psychosocial influence (parent support, parent pressure, sibling influence, coach support, one-to-one coaching time, peer influence) | 42 (30:12)  **Expert** (international senior-level competition; *M*=30.2, *SD*=4.9)  **Non-experts** (high-level junior participation only in European  or World junior championships; *M*=29.1, *SD*=3.8)  Soccer, Volleyball, Handball, Rowing, Swimming, Judo; | Non-experts revealed a significantly earlier investment in becoming elite athletes. No difference between groups for number of sports they took part in during developmental years | Non-experts invested earlier, suggesting that delayed investment is better (**tenet #7**) |
| Bridge, Toms;  2013;  UK | Identify whether early specialisation or sport sampling throughout childhood and adolescence influences performance levels during the investment phase of the DMSP, prior to adulthood | Cross-sectional;  Retrospective sport participation history | 1006 (463:543)  Highest standard of competition between 16-18 years: None, school, club, representative, national  Age at data collection*: M*=26, *SD*=6  Various; | Compared to playing in less sports, taking part in at least 2 sports from age 11 to 15 years increased the likelihood of playing on a representative/national team compared to lower levels of competition | An increased likelihood of achieving a higher standard of competition when individuals participate in three competitive sports during the specialising years of the DMSP model and supports the pattern of sport sampling proposed in the model (**tenet #2, #6,#7**) |
| Coutinho, Mesquita, Davids, Fonseca, Côté;  2016;  Portugal | Characterize the developmental pathways of highly skilled and less skilled volleyball players | Mixed-methods;  Quantitative: Retrospective sport participation history  Qualitative: main questions focused directly on developmental sport activities | 60 (30:30)  **High skill** (playing in premier league or senior national; all participants ≥ 23)  **Low skill** (recreational volleyball, never ben part of a senior national team; all participants ≥ 23)  Volleyball; | Volleyball players followed patterns consistent with DMSP sampling/ specialising/ investing years; high skill and less skill players reported about the same type of activities, but high skill players spent more hrs structured practice | These findings are consistent with the tenets of the DMSP that suggests two sport participation trajectories related to talent development: 1) elite performance through early diversification and deliberate play, and 2) elite performance through early specailization and deliberate practice (**tenets #1, #2, #6, #7**) |
| Coutinho, Mesquita, Fonseca, Martin-Silva;  2014;  Portugal | Characterize sport participation of Portuguese volleyball players at different and to determine if sport participation is different between level of expertise and gender | Cross-sectional;  Retrospective sport participation history | 229 (143:86)  **Skilled players** (highest level of Portuguese volleyball; all participants ≥ 19)  **Less skilled** (playing in second league; all participants ≥ 19)  Volleyball | Early or late specialisation does not hinder success in volleyball; skilled players accumulated more hrs than less skilled players, but did not perform more sports; males practiced more sports, more team sports and more competitions than females; skilled females practiced more individual sports than all others during stage 1 (6-12 years) and 2 (13-14 years); | While the quantity of practice emerged as a requisite to expertise achievement in this study, both early specialization and early diversification seem to be a factor that did not narrow or hinder the high-level achievement of our participants (**tenets #1, #7**) |
| Cupples, O'Connor, Cobley;  2018;  Australia | Examined the behavioural developmental trajectories of a large sample of professional Australian national rugby league players | Cross-sectional;  Retrospective sport participation history | 224 (224:0)  **Professional** (mean game experience = 68; range = 0-260;  *M*=25.6, *SD*=3.6)  Rugby; | Participants increased their involvements in other sports from 5-12 years, stabilised between 10-16 and reduced at 17+ | Present findings identified no evidence of any athletes adhering to an "early specialisation" trajectory. Rather, trajectories shared behavioural patterns adhering to DMSP sampling-specialising-investment pathway as both groups transitioned through similar stages (**tenets #1, #6, #7**) |
| Forsman, Blomqvist, Davids, Konttinen, Liukkonen;  2016;  Finland | Understand the role of sport-specific play and practice in the development of adolescent team sport athletes in the Finnish sports development system | Cross-sectional;  Retrospective sport participation history | 441 (441:0)  **National youth team athletes**  **Other**  *M*=15.3, *SD*=0.3  Soccer, ice hockey, basketball; | Sport specific play and practice undertaken during childhood were related to time spent in sport specific play and practice during early adolescence, and is associated to technical, tactical, and psychological skills; more experience with sport specific play and practice increased odds of being selected to national youth teams | Finnish developing athletes who reported higher amounts of sport-specific play and practice during childhood, also practised their primary sports more during adolescence (**tenet #4**) |
| Fraser-Thomas, Côté, Deakin;  2008;  Canada | Investigate physical and psychosocial developmental factors associated with sport dropout and engagement | Cross-sectional;  Retrospective sport participation history psychosocial (parents, coaches, peers, siblings) | 50 (8:42)  **Engaged** (11 regional, 14 provincial/national; *M*=18.7, *SD*=6.6)  **Dropout** (i.e. withdrawal between the ages of 14 and 17, within the past three years; 12 regional, 13 provincial/national; *M*=17.6, *SD*=4.1)  Swimming | Dropouts participated in less unstructured play than engaged athletes; dropouts started dry land, training camp, and were considered “top in club” earlier. Fewer dropouts took time off | Dropouts demonstrated a clear pattern of early specialization; early sampling is favorable (**tenet #2**) |
| Gallant, O'Loughlin, Brunet, Sabiston, Bélanger;  2017;  Canada | Describe naturally occurring sport participation during childhood and adolescence and determine if childhood sport profile predicts sport profile in adolescence | Longitudinal cohort;  DMSP-based categorisations following self-report organised and unorganised PA | 756 (340:416)  Participants self-reported involvement in 36 different PA that were then categorized as organized or unorganized (*M*=10.7, *SD*=0.6) | Early sport specialization did not predict against sport non-participation at mid-adolescence, whereas early sport sampling did | Early sport samplers were less likely to later become nonparticipants (**tenet #2**) |
| Ginsburg, Smith, Danforth, Ceranoglu, Durant, Kamin, Babcock, Robin, Masek;  2014;  USA | Explore patterns of specialization in a sample of professional minor league baseball players | Cross-sectional;  Retrospective sport participation history | 708 (708:0)  **Professional minor league baseball** (*M*=22.9, range: 18-39)  Baseball | players who attended college specialized later (16 vs 12 yrs); All participants played competitive baseball at all age ranges;  Players sampled until 15-18, 94% only played baseball at 19; 52% did not specialize until at least 17;  Players living in year-round climates were more likely to specialize early | Most athletes in the current sample began playing sports at approximately age six, but did not devote their time exclusively to a single sport until late into adolescence (**tenet #1,#6,#7**) |
| Güllich;  2014;  Germany | Examine the developmental activities of the 2012 Olympic champion men’s field hockey team | Cross-sectional;  Retrospective sport participation history | 54 (54:0)  **Olympic champions** (2012; n=16, *M*=26.6, *SD*=3.1)  **National success** (2012; n=19, *M*=22.3, *SD*=3.6)  **World class** (2002;n=19, *M*=24.2, *SD*=2.6)  Field hockey; | Olympic champions started sports involvement during early childhood, performed moderate amounts of sport-specific practice/training throughout their career, participated extensively in other sports, specialised relatively late and engaged over very long periods before eventually attaining international senior medals; they differed from national class in more extensive organised participation in various sports and later specialisation | From a conceptual perspective, each of the sub-samples exhibited some correspondence with as well as deviation from either pathway model in DMSP at a descriptive level, Older age ranged than predicted in DMSP (**tenet #7**). |
| Güllich;  2017;  Germany | Examine developmental participation patterns of international top athletes (medallists vs. non-medallists) | Match-paired design;  Retrospective sport participation history | 166 (86:80)  **National German squad** (Olympic and World champions, Olympic/world silver/bronze, European champions)  *M*=25.0, *SD*=4.7  Olympic sports; | Medallists started career in another sport and had experienced organised practice and competition in other sports before changing to their main sport;  Medallists engaged in organised practice/training and competitions in other sports considerably longer periods and specialised later than non medallists;  Different sports varied notably in total main sport practice/training volume; but no differences between success level and volume | Both groups mostly corresponded to early diversification, early specialization was underrepresented among medallists (**tenet #1**). Refinements to predictions of DMSP, regarding the particular significance of organised other-sports involvement, maintaining this involvement until late adolescence or beyond (**tenet #6, #7**) |
| Güllich;  2018;  Germany | Compare developmental participation patterns of elite track and field athletes with greater and lesser multi year performance improvement | Cross-sectional;  matched pairs design; paired for gender, track and field discipline and baseline level of performance;  Retrospective sport participation history;  Motives for participation in competitive sports | 218 (170:48)  **Junior** (age 13-17 years)  Strong responders (M=21.6, SD=4.7)  Weak responders (M=22.2, SD=5.0)  **Senior** (age 19-23+ years)  Strong responders (M=26.3, SD=3.1)  Weak responders (26.2, SD=3.7)  German national athletics association; | strong responders were more likely to experience practice and competition in other sports before entering athletics, and accumulated more organised practice and competitions in athletics from 14 to 17 than weak responders; strong responders continued to improve in performance over more years | The findings are consistent with numerous retrospective studies demonstrating that early intensified sport specific practice promotes rapid attainment of juvenile performance (**tenets #6,#7**) |
| Güllich;  2019;  Germany | Examine developmental participation patterns of female world-class and national-class football players | Cross-sectional;  Retrospective sport participation history | 29 (0:29)  German Bundesliga  **National team players** (n=14); *M*=25.1, *SD*=2.3  **Professional players** (n=15); *M*=24.1, *SD*=2.4  Soccer | National team players started peer-led football play younger and maintained play in peer led football and coach led practice in other sports longer;  National team players performed significantly less physical conditioning through 11-25 and significantly greater playing 15-25;  Group membership was predicted by 3 variables: proportion of playing forms within coach-led football practice; years of engagement in peer-led football play; accumulated coach-led practice hours in other sports | Childhood/ adolescent coach-led practice, but not peer-led play, in other sports differentiated senior performance. Furthermore, participation in other sports and peer-led football persisted to considerably later ages than predicted by the DMSP (**tenet #6, #7**) |
| Güllich, Emrich;  2014;  Germany | Examining athletic biographies of German elite athletes | Cross-sectional;  Retrospective participation histories (3 sub-studies)  1: retrospective comparison of successful/less successful junior and senior age athletes  2: longitudinal assessment 3 years later  3: comparison between types of sports and combinations of practice sports | 1558 (888:758)  Study 1: n=1558; Study 2: n=244; Study 3: n=1347  At the time of data collection:  55% **junior international competition** (*M*=17.1, *SD*=1.6)  45% **senior** **international competition** (*M*=24.4, *SD*=4.8) | Early training and competition in sport is associated with early success in sport, but not later success in sport;  The proportion of world class athletes participating in other sports was higher than within national class through all age periods, and world class athletes joined their main sport later | The absence of data suggesting a superiority of an earlier specialisation for long-term attainment of senior WC success in any sports category (**tenet #1**); The beneficial effect of sports-spanning variability of involvement not only during childhood but also during adolescence and adulthood (**tenet #6, #7**) |
| Güllich, Kovar, Zart, Reimann;  2017;  Germany | Examine contributions of different types of sport activities to the development of elite youth soccer performance | Longitudinal (2 time points 2 years apart)  Retrospective sport participation history  match-play (5v5 small sided game, rated on 7 match play attributes by experts) | 44 (44:0)  **Youth soccer academy** (highest age-level regional league; n=17, *M*=11.1, *SD*=0.4 (T1))  **Other clubs within the same league** (n=27, *M*=11.1, *SD*=0.4 (T1))  Soccer | Strong responders spent more time doing other activities in other sports than weak responders (sport sampling);  Performance at T1, and different activity types performed before T1 and between T2 explained 65% of variance of performance at T2; | The present findings suggest a delayed effect in that the combined activities did not differentiate performance at T1, but only at T2; while activities performed exclusively before T1 contributed to later performance differences at T2 (**tenet #5**) |
| Haugaasen, Toering, Jordet;  2014;  Norway | Identify the development of engagement in football specific activities of elite youth soccer players | Comparative research design;  retrospective practice history questionnaire;  two additions: 1. each activity into separate chronological age categories (play for all before coach-led), 2. players were specifically instructed to include all football specific school practice. | 543 (543:0)  All members of Norwegian Premier League (U-21)  **Professional** (senior professional contract; *M*=18.7, *SD*=1.3)  **Non professional** (no contract;  *M*=15.8, *SD*=1.6)  Soccer | 90% of the sample had started playing soccer by age 6 yrs; Professionals had accumulated about 20% more hrs of practice from ages 6-12 years and more accumulated by 19, but this was non-significant;  Professionals reported a greater contribution of activities with deliberate intentions (attacking, defending, physical mental) than non professionals; | It could therefore be that the differences in participation at the youngest age categories have provided the professional players with a motoric and cognitive advantage for future learning and performance, compared to the non-professionals (**tenet #5**) |
| Hayman, Polman, Taylor, Hemmings, Borkoles;  2011;  UK | Examine the nature of developmental, psychosocial and contextual factors experienced by adolescent golfers | Qualitative;  Retrospective sport participation history and psychosocial influences throughout development | 8 (8:0)  **U18 national** (*M*=18.8, *SD* = 2.1)  **England Senior A team development** (*M*=18.8, *SD* = 2.1)  **Senior men’s A team** (*M*=18.8, *SD* = 2.1)  Golf; | Golfers did not specialise in golf until it became their sole purpose in life around 16 years;  Golfers played several team and individual sports: during the sampling yrs, they played 4.8 sports; during the specializing years they played 4.5 sports; and during the investment years 1.8 sports; age of first golf involvement around age 9.5 yrs, first competition competition at age 11.1 yrs | Young aspiring sports performers up to the age of approximately 16 should experience multiple sports within a non competitive, task orientated, fun based learning environment that facilitates the refinement of motor skills as opposed to deliberate practice (**tenets #1, #7**) |
| Hendry, Crocker, Hodges;  2014;  Canada* data from UK | Determine whether the amount, or relative amounts, of soccer play and practice during childhood are associated with self-determined motivation among elite soccer players | Cross-sectional;  Retrospective sport participation history;  Motivation: BRSQ | 144 (144:0)  **U13** (12.0-12.9 years)  **U15** (14.0-14.9 years)  **U17** (15.0-16.9 years)  Soccer | Play hours during childhood were not associated with current levels of intrinsic or autonomous motivation. Prolonged exposure to the academy environment showed potentially detrimental relationships with motivation | We were unable to support the hypothesis that hours spent in play during childhood will positively relate to current levels of motivation among elite athletes who had either just finished childhood or were nearing adulthood (**tenet #4**) |
| Hendry, Crocker, Williams, Hodges;  2019;  UK | Determine if self-determined motivation in elite, men's soccer changes over time and differs as a function of age, skill-grouping, and engagement in soccer play and practice | Prospective;  T1 (2011; elite only)  T2 (2014; elite + non-elite);  Measures: Motivation, soccer specific, and Behavioral Regulation in Sport (BRSQ: 4 item subscales to measure six behavioral regulations from SDT); | 63 (63:0)  **Elite** (professional youth U15(n=15) & U17(n=16) academy Scotland)  **Non-elite** (age groupers Canada regional U15(n=16), U17 level(n=16))  Soccer | In elites, autonomous motivation decreased from T1 to T2 from U15 to U17; elites scored higher for SDT and autonomous motivation than non-elites | Data does not support idea that engaging in childhood play would foster later intrinsic and self-determined motivation (**tenet #4**) |
| Hendry, Williams, Ford, Hodges;  2019;  UK,  Canadian athletes | Assess developmental activities that best define elite female soccer players. | Cross-sectional;  Retrospective sport participation history | 45 (0:45)  **National team** (*M*=28.3, *SD*=4.0)  **Varsity team** (*M*=19.6, *SD* = 1.3)  Soccer; | National level players accumulated more hrs in soccer play than Varsity players, and National players rated practice and play as being moderate to high in challenge compared to their current skill level | Inconsistent with both DMSP pathways is the result that multisport participation increased from childhood to adolescence (**tenet #6, #7**) |
| Horning, Aust, Güllich;  2016;  Germany | Examine the developmental activities of German soccer players | Cross-sectional;  Retrospective sport participation history | 102 (102:0)  **Professionals** (1^st^ Bundesliga; including National Team, n=52, *M*=30.7, *SD*=4.2)  **Senior amateurs** (3^rd^-6^th^ leagues; top 2% of all German senior amateur players, n=50, *M*=24.8, *SD*=4.1)  Soccer | Professional players specialised later and performed more leisure football in childhood, more sports in adolescence and more organised football in adulthood than amateurs;  Most players' developmental participation patterns corresponded only partly to and partly deviated from the attributes of either pathway model described in DMSP | Also, most footballers continued engagement in leisure football and in other sports significantly longer than predicted by DMSP (**tenet #7**), and their development was characterised by continuous gradual increase or decrease of the respective activities rather than reflecting transition points between distinguishable stages |
| Huxley, O'Connor, Bennie;  2018;  Australia | Investigate factors during the specialising and investment stages that underpinned development of elite senior athletes | Qualitative;  Semi-structured interviews: athlete developmental experiences during the specialising and investment stages in relation to (1) training and competition; (2) psychosocial support and (3) psychological factors | 14 (7:7)  **Senior elite** (represent country at Olympic Games and/or World Championships; *M*=35.6, *SD*=7.34)  Track and field | Young athletes are not physically or psychosocially ready to enter the investment stage at least age 16 years | most were late specialisers and did not focus on main even until end of investment stage (**tenet #7**) |
| Huxley, O'Connor, Larkin;  2017;  Australia | Retrospectively examine developmental influences, milestones and specific competition pathways of elite senior Australian Track and Field athletes | Cross-sectional;  Retrospective sport participation history | 73 (39:34)  **Senior elite** (international level for an average of 8.5 yrs; *M*=51, age range=23-87)  Track and field; | Most athletes (63,8%) specialized between 12 and 17;  Six pathways were identified within 5 competition levels; Specialising later and remaining involved in other sports in late adolescence was conducive to later success;  importance of support from families and coaches/teachers is discussed | Many athletes in the study with the majority achieving global level of competition via the sampling route (**tenet #1**). |
| McFadden, Bean, Fortier, Post;  2016;  Canada | Investigate the psychological effects of sport specialization by examining relationships between youth hockey players’ level of specialization and psychological outcomes | Cross-sectional;  retrospective sport participation history  Mental Health Continuum-Short Form; mental illness; Psychological need satisfaction, Psychological need dissatisfaction | 61 (61:0)  **Bantam/*M*idget hockey players**;  Early specializers (n=26 *M*=15.1, *SD*=1.4)  Late specializers (n=15, *M*=14.4, *SD*=1.5)  Recreational (n=20, *M*=15.1, *SD*=1.3) | Early specializers had highest psychological need dissatisfaction and recreational athletes had the lowest psychological need dissatisfaction | Results support DMSP in that early sport specialization may lead to higher psychological need dissatisfaction; specializing after age 12 may be favourable without compromising performance (**tenet #6**) |
| Mendes, Nascimento, Souza, Collet, Milistetd, Côté, Carvalho;  2018;  Brazil | Examine the patterns of cumulated structured practice and other structured sports activities of elite adult Brazilian volleyball players | Cross-sectional;  Retrospective sport participation history | 78 (78:0)  **Elite** (National team, n=14, *M*=19.2, *SD*=0.9)  **Non-elite** (Highest level of Brazilian volleyball, but not selected on national team, n=64, *M*=19.2, *SD*=0.9)  Volleyball; | Overall players spent more time in other sports than volleyball. Earlier specialization in volleyball was associated with greater volumes of volleyball hours; no difference in accumulated practice was apparent between players that reached international level | The present results with elite volleyball players display the possibility of a positive relation between diversified sport practice and expertise in sport (**tenet #1**). A trend of late specialization in volleyball was observed and despite large between players variation, no differences  in the trend of accumulated structured practice were apparent between players that reached international level (**tenet #7**) |
| Moesch, Elbe, Hauge, Wilkman;  2011;  Denmark | Investigate group differences concerning accumulated practice hours during development in elite and near-elite athletes | Cross-sectional;  Retrospective sport participation history | 242 (147:95)  Team Danmark  **Elite** (top 10 World or European medal, *M*=24.5, *SD*=7.5)  **Near-elite** (not elite, *M*=24.5, *SD*=7.5)  Centimeters, Grams, & Seconds sports | Elite athletes completed more training hrs after age 18 yrs; near elites completed more training hrs before 18;  Elites reached milestones in career later, and spent fewer years on junior national team but more years on senior national; spending less time on junior National team, one additional year on senior national team, less practice hrs at age 15 yrs, and more practice hrs at age 18 yrs significantly predict international success | The findings of the study confirm the idea of late specialization of one sport (**tenet #7**), as suggested in the early diversification path postulated by DMSP, however, results concerning involvement in different sports do not confirm the proposed advantage of sampling several sport experiences for CGS athletes (**tenet #1)** |
| Sieghartsleitner, Zuber, Zibung, Conzelmann;  2018;  Switzerland | Investigate developmental differences of Swiss junior national soccer team players compared to less successful peers | Cross-sectional;  Retrospective sport participation history | 294 (294:0)  **National players** (at least one nomination for Swiss Junior National Team (U15-U18; n=57)  **Regional players** (Passed regional squad selection; no SJNT; n=183)  **Local players** (Failed regional squad selection; n=54)  Soccer | 5 clusters of players were identified: football enthusiasts, club players, average players, poly-sportive players; football abstainers;  Football enthusiasts and club players had increased chance to get selected to the Swiss junior national team;  Poly-sportive players tend to reach an average regional level; Swiss soccer follows a specialized sampling model | Football enthusiasts (most accumulated hrs free play) had the highest likelihood of achieving high playing standard (**tenet #5)**.  The presented findings support previous work showing the essential role of sport participation up to 12 years of age (**tenet #1)** |
| Soberlak, Coté;  2003;  Canada | Investigate the developmental activities of professional ice hockey players | Cross-sectional;  Retrospective sport participation history | 4 (4:0)  **Elite** (Canadian Hockey League players who signed professional contracts in National Hockey League; *M*=20)  Ice hockey | Athletes achieved expert status without significant involvement in the early years. They decreased involvement in deliberate play and increased involvement in deliberate practice around age 13-15 years | As athletes progress from sampling to investment years, involvement in deliberate play decreases while involvement in deliberate practice increases (**tenet #6**) |
| Thomas, Güllich;  2019;  Germany | Empirically test the postulate that early diversification will beneficially affect the future intrinsic motivation among elite youth athletes | Cross-sectional;  Retrospective sport participation history  Sport Motivation Scale | 178 (132:46)  **Federate state youth squad** (n=78, *M*=15.3, *SD*=1.5)  **National youth squad** (n=39, *M*=15.3, *SD*=1.5)  Badminton, cycling, football, judo, tennis | Main sport start at age 6.4 yrs, first competition age 7.5 yrs, start sport school age 11.5 yrs; Results did not provide support that early sampling leads to higher adolescent intrinsic motivation | The results did not provide evidence that early diversification leads to higher levels of adolescent intrinsic motivation (**tenet #4)** |
| Vink, Raudsepp, Kais;  2015;  Estonia | Examine prospective associations between intrinsic motivation and individual deliberate practice in specializing team sport athletes | Longitudinal cohort (3 times between 2010/11-2011/12 season; beginning and end first season, start 2^nd^ season);  Retrospective sport participation history  Sport Motivation Scale | 163 (102:61)  **Youth championship** (*M*=13.6, *SD*=0.3) | Both intrinsic motivation and deliberate practice increased over 1 year;  Greater intrinsic motivation influences greater deliberate practice and deliberate practice levels increase intrinsic motivation | Results demonstrated associations with intrinsic motivation and deliberate practice hrs in sample of specializing adolescent athletes (**tenet #4)** |

| Appendix 2b. Extracted data from the studies that supported tenets of the Long-Term Athlete Development Model | | | | | |
| --- | --- | --- | --- | --- | --- |
| Authors;  Year;  Country | Major focus of the study | Study design;  Measures | n (male:female)  **Level of competition** (description; age)  Sport(s) | Main findings | Tenet(s) tested |
| Arede, Esteves, Ferreira, Sampaio, Leite;  2019;  Portugal | Identify the relationship between previous sport experiences, maturational and physical characteristics of youth basketball players | Longitudinal (2 time points, 1 year apart)  Retrospective  sport participation  history anthropometrics data, Maturity offset (leg length + Age from PHV), lower body power, speed | 68 (36:32)  Selection for **U14 national team** (*M_M_*=12.19, *SD_M_*=0.58; *M*_F_=12.03, *SD*_F_=0.54)  Basketball; | Less specialized athletes started basketball involvement later than more specialized subjects; More specialized athletes reported more basketball deliberate practice and free practice; Less specialized athletes performed better on physical parameters | The results seem to suggest that the less specialized path was beneficial for sprinting and jumping capacities. Also, diversified and non-specific early sport involvement (**tenet of specialization)** seem to have a beneficial effect on jumping and sprinting performance |
| Krisiansen, MacIntosh, Parent, Houlihan;  2018;  Norway | Examine the impact of participation in the Youth Olympic Games for athletes that have continued or dropped out | Cross-sectional;  Instrument captured quantitative and qualitative data for 6 areas (performance and experience, education, leaving sport, pursuing an elite career, learning and legacy) | 68 (34:24)  **Team Norway, Youth Olympic Games** (age not described in study) | The majority of respondents were still involved in elite-level sport, although a substantial minority, 29% (n = 17), had dropped out.  Seven of the 17 medal winners within the study were among the group of dropouts; suggesting that winning a medal did not seem to be important in determining whether an athlete stayed in elite sport or not. | The national context, in terms of the sport and education systems, must align to ensure young athletes do not need to choose between elite-level sport and school. The national context may play a larger role in deeper engagement in sport than YOG participation/success (**tenet of alignment)** |
| McCunn, Weston, Hill, Johnston, Gibson;  2017;  Germany | Investigate the influence of relative age on physical maturity and sprinting speed | Repeated cross sectional (8 years);  Anthropometric measures, physical performance tests; relative age effect, Maturity offset, sprint performance | 306 (306:0)  **Scottish Premiership club academy** (age range: 9.7-16.6 years)  Soccer | Clear relative age effects across age groups were found and physical maturity was related to chronological age, especially at the U-14 and U-15 age categories | At the under-14 and under-15 age categories, relatively older players were generally more mature and this manifested as faster sprinting speed (**tenet of age)** |
| Moran, Parry, Lewis, Collison, Rumpf, Sandercock;  2018;  UK | Assess the effects of speed training on sprint speed before (Pre-PHV) and during (Mid-PHV) the growth spurt in youth soccer players | Quasi-experimental (1 speed training/wk for 8wk);  Maturity offset, test performance | 42 (42:0)  **English Professional category three academy** (second lowest level)  Pre-PHV (n=12 experimental; n=13 control; age range: 10-10.4 years)  Mid-PHV (n=7 experimental; n=10 control; age range: 13.6-14.5 yrs)  Soccer | The applied dose of speed training was more effective in the Pre-PHV group than in the Mid-PHV group | **Tenet of trainability**: which states that windows of opportunity exist for accelerated development of certain abilities |
| Yustres, del Cerro, Martin, Gonzalez-Mohino, Logan, Gonzalez-Ravé;  2019;  Spain | Provide a detailed, swimming-specific approach to examining the influence of early specialization in elite swimmers. | Observational retrospective study;  Used historical data from official results websites from 2007, 2009, 2011, 2013, 2015, 2016 WC, and 2006, 2008, 2011, 2013, 2015, 2017 junior WC;  Race performance | 4076  **Cat 1** (participated in WC without junior WC; n=3371 (1757:1614))  **Cat 2** (only junior WC)  **Cat 3** (participate in WC AND junior WC; n=705(388:317))  Swimming | Although the ratio of conversion (17.29%) from junior to senior was low, the results showed that swimmers that had previously participated in junior WCs reached 9.01 better positions than Cat 1 swimmers in senior WCs. | Early specialization is a significant factor to achieve better results in senior categories. 40% who transfer from junior end up in finals during senior (**tenet of specialization)** |
